# *NorthStar*, a support tool for the design and evaluation of quality improvement interventions in healthcare

**DOI:** 10.1186/1748-5908-2-19

**Published:** 2007-06-26

**Authors:** Elie A Akl, Shaun Treweek, Robbie Foy, Jill Francis, Andrew D Oxman, the ReBEQI group

**Affiliations:** 1 Department of Medicine, State University of New York at Buffalo, USA; 2 Tayside Centre for General Practice, Community Health Sciences Division, University of Dundee, UK; 3 Centre for Health Services Research, Newcastle University, UK; 4 Health Services Research Unit, University of Aberdeen, UK; 5 Norwegian Knowledge Centre for the Health Services, Oslo, Norway; 6 Research-Based Education and Quality Improvement, European partnership

## Abstract

**Background:**

The Research-Based Education and Quality Improvement (ReBEQI) European partnership aims to establish a framework and provide practical tools for the selection, implementation, and evaluation of quality improvement (QI) interventions. We describe the development and preliminary evaluation of the software tool *NorthStar*, a major product of the ReBEQI project.

**Methods:**

We focused the content of *NorthStar *on the design and evaluation of QI interventions. A lead individual from the ReBEQI group drafted each section, and at least two other group members reviewed it. The content is based on published literature, as well as material developed by the ReBEQI group. We developed the software in both a Microsoft Windows HTML help system version and a web-based version. In a preliminary evaluation, we surveyed 33 potential users about the acceptability and perceived utility of *NorthStar*.

**Results:**

*NorthStar *consists of 18 sections covering the design and evaluation of QI interventions. The major focus of the intervention design sections is on how to identify determinants of practice (factors affecting practice patterns), while the major focus of the intervention evaluation sections is on how to design a cluster randomised trial. The two versions of the software can be transferred by email or CD, and are available for download from the internet. The software offers easy navigation and various functions to access the content. Potential users (55% response rate) reported above-moderate levels of confidence in carrying out QI research related tasks if using NorthStar, particularly when developing a protocol for a cluster randomised trial

**Conclusion:**

*NorthStar *is an integrated, accessible, practical, and acceptable tool to assist developers and evaluators of QI interventions.

## Background

Clinical evidence is essential to enhance the delivery of care and improve patient outcomes. Yet the transfer of research findings into clinical practice tends to be a slow and haphazard process. One of the central aims of quality improvement (QI) activities is to enhance such transfer. In spite of the growing body of rigorously conducted studies on the effectiveness of various QI strategies [1], the strategies selected for QI activities tend to be based more upon prevailing disciplinary customs and habits than scientific evidence and explicit rationales [2,3].

In 1999, the European Commission (EC) published a report that considered various ways to improve the effectiveness and efficiency of European health care systems [4]. The report recommended work to inform strategies for the effective dissemination and implementation of research findings. The Research-Based Education and Quality Improvement (ReBEQI) project was developed to improve QI research and practice, and respond to the EC recommendations [5]. The project group comprised seven partners in six European countries (Appendix, see Table 4), each with substantial experience in QI research [6]. The project took place over three years (December 2002 – November 2005) and was funded by the European Commission's Quality of Life and Management Living Resources 5 th Framework Program. The ReBEQI project had two objectives:

1. To establish a framework for the selection, implementation, and evaluation of interventions to promote evidence-based clinical practice.

2. To provide clinicians, policy-makers, and researchers with a suite of evidence-based, Internet tools, guided by the framework, to inform the development and evaluation of interventions to promote evidence-based clinical practice.

*NorthStar *is a major product of the ReBEQI project. It is a software program that packages information on the design and evaluation of evidence-based QI interventions into an integrated, easily accessible, and practical tool. In this paper, we provide a general overview of the guiding principles, content development and software development of *NorthStar *and report its preliminary evaluation. We refer readers to the ReBEQI website for further information on ReBEQI and to see the NorthStar content in full [5].

## Methods

### Guiding principles

The development of *NorthStar *was guided by four principles. First, the information and tools it offers need to be evidence-based, while being explicit about the limitations of existing evidence. For example, whilst there is a growing body of evidence on the effectiveness of various QI interventions, it is recognised that no single intervention will be consistently effective across a wide range of problems and contexts [1]. Second, *NorthStar *has to be appropriate to the diverse needs of its target audience, who may have varying levels of skills and experience in quality improvement and research. Third, *NorthStar *has to be simple to read and navigate to ensure its relevance, acceptability and usefulness.

### Content development

The target audience of the tool include QI researchers, healthcare professionals and managers responsible for developing, delivering and evaluating continuing education (CE) and QI programmes at a national or regional level. Consequently, the two areas we covered were the design of QI interventions and their evaluation. The intention was for the tool to be useful to users with varying levels of experience in QI research, and for the text to be instructional as well as informative. For example, when describing the different types of cluster, randomised controlled trials (cluster RCTs), in addition to outlining the features of each type, the text would guide the user with regard to when the use of each design might be appropriate.

A lead individual from the ReBEQI group was identified for each section of the tool according to individual expertise. This lead person took responsibility for preparing the first draft of that section, based on a review of the published literature as well as other outputs of the ReBEQI project (e.g. a literature review of QI models and frameworks). At least two other ReBEQI members reviewed the section. An editor ensured that the different sections were consistent with regard to content, structure and writing style. All group members reviewed the final version for wider feedback and final approval.

The content builds on the framework developed by ReBEQI for the selection, implementation and evaluation of QI interventions. It guides the user on how to identify "determinants of practice" in designing the intervention and on how to design a cluster RCT as the preferred evaluation study design. Determinants of practice are the factors that affect practice patterns and explain the variation in the effectiveness of different QI interventions in changing practice patterns; they are usually categorized as "barriers and facilitators" or as "moderators and mediators" [7,8]. The *NorthStar *section on cluster RCTs is based, in part, on the Trial Protocol Tool, a product of the Pragmatic Randomized Controlled Trials in HealthCare (PRACTIHC) group [9].

### Software development

We developed *NorthStar *as a Microsoft Windows HTML help system, the standard help system used with Windows 95 and above. This choice gives the tool a familiar Windows interface and provides a range of off-the-shelf functionality. The software for running Windows HTML help, including Internet Explorer upon which it relies, is part of the Windows operating system. The basic HTML help development tool, Microsoft HTML Help Workshop, is available free from Microsoft's website. We also created a web-based version using chm2web by A!K Research Labs [10] that is platform-independent and works with any modern browser.

For the content, we created HTML versions of Word documents using Mozilla 1.4 for Mac OS X. We then linked the HTML documents to the help system in HTML Help Workshop. The HTML documents can contain links to the full spectrum of electronic document formats and can, therefore, include not only textual information but graphics, documents created by other programs, sound, video, as well as links to websites and other programs. Once the HTML documents have been added or updated, the collection is compiled into a single file (a .chm file). The compiled .chm file included the main text of *NorthStar*, while accompanying documents such as pdf or Word files were organized as external files in the same folder as the compiled .chm file.

### Preliminary evaluation

We undertook a questionnaire survey of two groups of potential *NorthStar *users. The first group comprised eight postgraduate students attending a research methodology course in Göteborg, Sweden. The course focused on the planning of intervention studies within the broader context of health systems research. The second group consisted of 25 individuals, nominated by the seven ReBEQI partners, responsible for developing, delivering, and/or evaluating CE and QI activities. Most of them were based in the ReBEQI partner countries. We surveyed the first group during a class session in 2006, and contacted the second group by e-mail during 2005–2006. We sent non-responders one e-mail reminder two weeks after the initial contact.

We asked respondents to spend around twenty minutes browsing *NorthStar *(with no constraints to the browsing sequence) and to fill out a questionnaire. First, we asked respondents about their main professional roles, their roles in CE and QI, and their experience in QI research. We then asked them to estimate the time spent browsing *NorthStar *and which sections they reviewed the longest. We also asked respondents to use a seven-point scale (1 representing 'not at all,' and 7 'a lot.') to rate their level of confidence with the use of *NorthStar*, and in carrying out 10 named tasks relevant to QI research. A final question requested open-ended feedback on the format and user-friendliness of *NorthStar*. A copy of the questionnaire is available as an Additional File. We used descriptive statistics to report aggregated responses from the two groups because of the relatively small number of participants from each.

## Results

### *NorthStar *Content

*NorthStar *consists of 18 sections covering the design and evaluation of QI interventions. After two introductory sections (About *NorthStar *and Introduction), *NorthStar *discusses how to involve stakeholders (Stakeholder Involvement). It then examines how to identify and select priorities in order to use resources efficiently (Priority Setting). Next, it provides guidance on how to identify, critically appraise, adapt, and develop relevant clinical practice guidelines (Clinical Practice Guidelines). It also discusses the why, what and how of measuring baseline performance defined as the measurement of actual clinical practice and its comparison to desired clinical practice (Measuring Baseline Performance).

*NorthStar *discusses what the determinants of practice are and how to identify them using both explorative methods and theory-based methods (Identifying Determinants of Practice). It then provides guidance on what intervention strategy to use, and how to select determinants of practice, and use them to tailor the intervention (Designing the Intervention).

*NorthStar *assists the user in the choice of appropriate study design to test the QI intervention (Choosing the Study Design). It then discusses the usefulness, limitations, and types of pilot studies (Pilot Studies). The major section of *NorthStar *is dedicated to the development of protocols for cluster RCTs (Cluster RCT Protocol Tool). Next, it examines when to use and how to design an interrupted times series evaluation (Interrupted Time Series Evaluation). *NorthStar *reviews two other types of evaluation (Process Evaluation and Economic Evaluation). It also provides guidance on how to form or identify networks of QI researchers, health professionals, health service managers, and policy makers (CE and QI programs). Finally *NorthStar *provides useful resources (Glossary, Libraries and Web Resources). Table 1 shows the general outline of each of these sections.

**Table 1:** The general outline of NorthStar sections

**This section contains the following**	An outline of the section
**Introduction**	One or two paragraphs introducing the topic
**Things to consider**	Checklists; background information; protocol considerations (relating to writing the trial protocol); and practical considerations (relating to implementing the intervention)
**Illustrative examples**	Examples taken from protocols or final reports of cluster RCTs of QI interventions. They show either different aspects of the topic or how different studies addressed the same aspect
**References**	Journal articles or books referenced in the text
**Resources**	Useful documents (accessible directly in NorthStar), links to websites, software, and recommended further reading (brief and sometimes annotated bibliography of key references)

### *NorthStar *software

**Figure 1:**
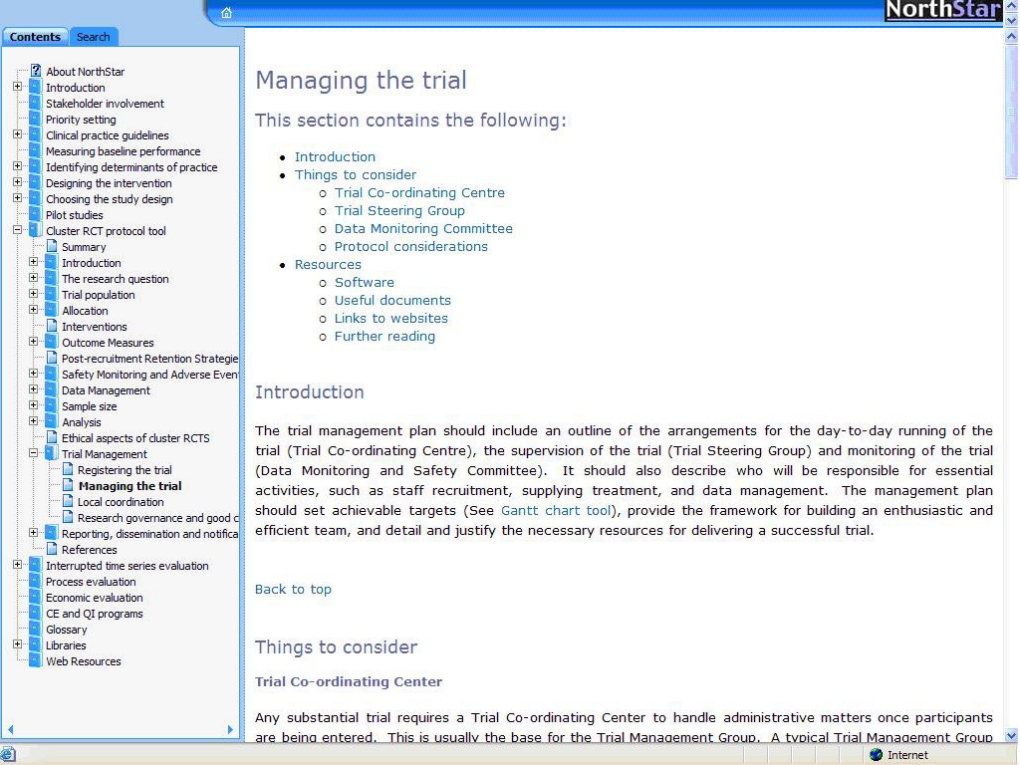
Screenshot from the browser-based version of NorthStar.

*NorthStar *is available as an HTML Help system, or as a browser-based version. Figure 1 shows a sample screenshot from the browser-based version of *NorthStar*. Both versions can be transferred by e-mail and on CD, and downloaded from the Internet [11]. Once downloaded, neither version of *NorthStar *requires an Internet connection for use, although links to websites do require a live Internet connection. The July 2006 version of the tool comprises a compiled .chm file (808 kB), an external set of libraries (9.2 Mb), and a collection of other supporting external material (6.8 Mb). Most of the main sections of *NorthStar *also are being translated into French and Italian.

The user can access the content of *NorthStar *in many different ways:

1. *Contents*: An outline at the left helps the user to locate what he/she needs. "Books" (each containing one of the 18 sections described above) can be expanded or collapsed by clicking on the +/- sign to the left of the book symbols.

2. *Search*: The user can search for words anywhere in the text.

3. *Hypertext*: The hypertext links help to navigate *NorthStar*, for example by bringing up relevant definitions or explanations in the glossary, examples or illustrations.

4. *Print*: Selecting 'Print' in the browser or from the Windows Help system will print the contents of the main *NorthStar *window.

5. *Libraries*: These can be accessed from the 'Libraries' menu at the top of the screen or from the bottom of the contents and include: checklists, an annotated bibliography of key methodological papers, structured abstracts of selected references, summaries of key theories, and examples of protocols and final reports of evaluations of QI interventions.

### *NorthStar *preliminary evaluation

Eight participants from the student group and ten from the ReBEQI nominees responded to the survey (total response rate 18/33; 55%). One respondent from the ReBEQI group provided free text responses only, so we included only 17 responses in the quantitative analysis.

Fourteen (82%) respondents described themselves as health care researchers; others were health care practitioners and managers. Nine (53%) reported intermediate levels of experience in QI research compared with seven (41%) who described themselves as either beginners or having no role in QI research and one (6%) experienced QI researcher. The mean time spent browsing *NorthStar *was 20.6 minutes. Participants spent most of the time looking at 'Identifying Determinants of Practice' (8/17; 47%) and 'Designing the Intervention' (6/17; 35%). The levels of confidence in carrying out, with the use of *NorthStar*, the ten tasks related to QI research, were all above the mid-point of the scale (i.e., four; Table 2). Respondents indicated the greatest confidence in being able to develop a protocol for a cluster randomised trial (mean 5.7) and the least confidence in being able to involve stakeholders in QI research and measuring baseline performance (both means 4.9).

**Table 2:** Respondents' self-rated confidence in carrying out tasks relevant to quality improvement research using NorthStar.

Task	Mean (range)
Involve stakeholders in quality improvement intervention research	4.9 (3–7)
Identify and select priorities in order to use resources efficiently	5.2 (4–6)
Critically appraise clinical guidelines	5.1 (4–7)
Measure baseline performance (defined as the measurement of actual clinical practice and its comparison to desired clinical practice)	4.9 (3–6)
Identify determinants of clinical practice	5.2 (4–6)
Decide what intervention strategy to use to improve the quality of care	5.4 (4–7)
Choose an appropriate study design to evaluate a continuing education or quality improvement strategy	5.5 (5–7)
Develop a protocol for a cluster randomised trial	5.7 (5–7)
Conduct a process evaluation	5.2 (4–6)
Design an interrupted time series evaluation	5.1 (4–6)

*Based on a 1–7 scale where 1 = not at all confident and 7 = very confident

Respondents generally made positive comments about the format and user-friendliness of *NorthStar *(illustrative comments in the Table 3). Some also mentioned technical problems around its use, while others questioned its major focus on RCTs as the 'gold standard' method for evaluating quality improvement strategies.

**Table 3:** Illustrative comments on user-friendliness, format and other features of NorthStar.

User-friendliness	*Very impressive in its scope and quality but it's not always clear who the audience for the tool is* *It is indeed a very beautiful tool I found it user friendly, and particularly liked the sample-size calculation module for cluster randomization* *Consider developing a more detailed tutorial (in parts) to walk individuals through how to use NorthStar to its fullest capability* *My computer crashed twice when trying to access pdf files*
Format	*[NorthStar] provides a clear overview, helps me think in a constructive and orderly fashion about what I need to do, and leaves me inspired*.
Content	*I can see the purpose in encouraging people as often as possible to conduct randomised trials of QI initiatives, but in fact most evaluation will not be done in this way, and I'm not sure that those seeking to use NorthStar for the less rigorous designs will be well served* *I think you risk misleading people with that stated aim [targeted at quality improvement researchers and healthcare professionals and managers] – it looks great for researchers, but not for anyone else* *Service quality improvers ... are unlikely towant to 'test' interventions in the formalsense, but they might want help with choosing interventions and testing their effect in their own particular contexts*

## Discussion

*NorthStar *is a tool that aims to support the development and evaluation of interventions to improve the quality of healthcare. Its target audience includes QI researchers, healthcare professionals, and managers responsible for developing, delivering and evaluating CE and QI programmes at a national or regional level.

*NorthStar *has a number of strengths. The work reflects the collective experience of several established European inter-disciplinary collaborations, but also is based on recent literature reviews and the ReBEQI group's own innovative methodological work. The development of the content employed iterative processes of drafting, reviewing and editing. The resulting content is simple to read, pragmatic and focused on assisting the user in developing and evaluating the QI intervention. In addition, *NorthStar *is available as both a browser-based version and an HTML help file version, and will soon be available in French and Italian, as well as English. While the focus of NorthStar is on QI programmes at a national or regional level, those in charge of smaller-scale programmes (e.g. local QI initiative) might also find selected components useful.

However, *NorthStar *has a number of limitations. Because of the limitations of the existing evidence, many recommendations within NorthStar are based on pragmatic interpretations of that evidence. We used structured rather than systematic reviews to draft sections – although these drew upon systematic reviews. In addition, we do intend to incorporate into *NorthStar *further evidence on the effectiveness of QI strategies as it becomes available.

The results of our modest evaluation suggest that *NorthStar *will form a useful reference and educational tool for QI research in general, and for developing a protocol for a cluster randomised trial, in particular. Potential users seemed mostly interested in the sections that identify determinants of practice and the design of interventions, which are both recognised as challenges requiring greater attention in the implementation field [12]. However, our evaluation of *NorthStar *was limited by a small sample size, the limited representativeness of participants, the lack of a comparison, and the assessment of intermediate outcomes. For example, we have not evaluated the impact of the use of *NorthStar *on the processes and outcomes of development and evaluation of QI interventions. It also is possible that actual or perceived technical barriers to using *NorthStar *will discourage some potential users.

## Conclusion

*NorthStar *is an integrated and practical tool to assist QI researchers, healthcare professionals, and managers responsible for developing, delivering and evaluating CE and QI programmes at a national or regional level. Our preliminary evaluation is broadly positive. In its current form, *NorthStar *is mainly designed to meet the needs of QI researchers.

## Availability and Requirements

### Project homepage



*NorthStar *is available from the 'Tools and databases' menu.

### Operating system

Windows HTML Help version works in Windows 95 and above.

Web-based system works with Linux, Mac OS 9/OS × and Windows.

### Other requirements

Windows HTML Help requires that Internet Explorer version 3 or above is installed. The browser-based version requires nothing more than a modern browser.

### License

ReBEQI is happy to have its products used for personal and non-commercial use and at no charge in free educational programmes. However, when tuition is charged, ReBEQI royalty fee is 10% of gross receipts. If you are planning to use *NorthStar *within a for-profit organisation in any way, please contact us to discuss a donation to our work.

## Competing interests

The author(s) declare that they have no competing interests.

## Authors' contributions

All authors participated in the development and evaluation of *NorthStar*. EAA and RF were the content editors. ST was responsible for the technical development of *NorthStar*. ADO was the central coordinator of the project. All authors read and approved the final manuscript.

**Table 4:** Countries, partner organisations and partner managers of the ReBEQI project

**France**: Santé Publique et Informatique Médicale (SPIM), Faculté de Médecine Broussais – Hôtel Dieu, Paris. Partner manager – Pierrre Durieux
**Italy**: Unit of Clinical Governance, Agenzia Sanitaria Regionale (Regional Health Care Agency) of Emilia-Romagna, Bologna. Partner manager – Roberto Grilli
**Italy**: Center for the Evaluation of Effectiveness of Health Care (Ce.V.E.A.S.), Modena. Partner manager – Alessandro Liberati
**The Netherlands**: Centre for Quality of Care Research, University of Nijmegen. Partner manager – Richard Grol
**Norway**: Norwegian Health Services Research Centre, Oslo. Partner manager – Andy Oxman
**Sweden**: Division of International Health, Department of Public Health Sciences, Karolinska Institutet, Stockholm. Partner manager – Cecilia Stålsby Lundborg
**United Kingdom**: Centre for Health Services Research, University of Newcastle, Newcastle. Partner manager – Martin Eccles
